# Negative mood and mind wandering increase long-range temporal correlations in attention fluctuations

**DOI:** 10.1371/journal.pone.0196907

**Published:** 2018-05-10

**Authors:** Mona Irrmischer, C. Natalie van der Wal, Huibert D. Mansvelder, Klaus Linkenkaer-Hansen

**Affiliations:** 1 Department of Integrative Neurophysiology, Center for Neurogenomics and Cognitive Research (CNCR), Amsterdam Neuroscience, VU Amsterdam, HV Amsterdam, Netherlands; 2 Vrije Universiteit (VU) Amsterdam, Department Computer Science, Amsterdam, Netherlands; 3 Centre for Decision Research, University of Leeds Business School, Leeds, United Kingdom; Consejo Nacional de Investigaciones Cientificas y Tecnicas, ARGENTINA

## Abstract

There is growing evidence that the intermittent nature of mind wandering episodes and mood have a pronounced influence on trial-to-trial variability in performance. Nevertheless, the temporal dynamics and significance of such lapses in attention remains inadequately understood. Here, we hypothesize that the dynamics of fluctuations in sustained attention between external and internal sources of information obey so-called critical-state dynamics, characterized by trial-to-trial dependencies with long-range temporal correlations. To test this, we performed behavioral investigations measuring reaction times in a visual sustained attention task and cued introspection in probe-caught reports of mind wandering. We show that trial-to-trial variability in reaction times exhibit long-range temporal correlations in agreement with the criticality hypothesis. Interestingly, we observed the fastest responses in subjects with the weakest long-range temporal correlations and show the vital effect of mind wandering and bad mood on this response variability. The implications of these results stress the importance of future research to increase focus on behavioral variability.

## Introduction

“Mind wandering” refers to cognitions unrelated to the current demands of the external environment and plays a prominent role in daily life [1]. Reflecting on the past or mentally simulating future events are key components of the mind wandering process and central to our self-percept [2,3]. Intrusive thoughts, however, may also cause attention lapses in vital situations demanding sustained attention such as driving [4]. Additionally, long-term stress and emotional burden can lead to an increase of mind wandering, often referred to as rumination [1,5], which has a relation to numerous brain disorders, including depression [6] and social anxiety [7], and is related to decreased task performance for example while reading [8].

Research on attention has a long tradition in investigating how we selectively process one source of information while ignoring others [9]. In the face of limited processing abilities, task performance is strongly influenced by the level of attention paid to the task [10]. Still, competing influences such as additional sensory information [11] or mind wandering [12] can cause distraction from the task at hand and play a pronounced role in determining behavioral variability, as seen for example in a metronome task requiring continuous responses to tones [13]. Nevertheless, most investigations into the link between attention and performance use statistical measures of central tendency, such as mean reaction times [14,15], and thereby ignoring large proportions of ongoing variability as noise or unexplained variance [16]. It has been shown that impending errors and stimulus-independent thoughts are related to increases in average reaction times [17,18] and reaction time variance [13,19,20], but the observation that behavioral responses have apparent fluctuations, which tend to occur in non-random clustered fashion [21,22] is largely neglected.

The lack of investigations into the temporal structure of trial-to-trial variability in reaction times is surprising considering the growing evidence that the temporal structure of neuronal and behavioral time series contains valuable information about the mechanisms and functions of the systems producing these fluctuations. In fact, if a signal exhibits complex fluctuations, the average is a poor statistical measure as opposed to scaling techniques that relate different scales to each other [23], especially if the signal harbors the long-term memory process known as 1/*f* noise [16]. 1/*f* noise is a hallmark of dynamical systems operating close to the critical point of a phase transition from ordered to disordered states [24] and is also a characteristic of many healthy physiological systems with high demands for swift adaptation, e.g., heartbeat [25], gait [26], speech [27] and neuronal oscillations [28,29]. In such non-linear dynamical systems operating near the critical state, these dynamics are reflected in the form of long-range temporal correlations [30–32]. Indeed, behavioral experiments in humans also show robust non-random clustering of behavioral responses such as detection of weak tactile [33] or visual stimuli [34], as well as in speech [35] and motor timing tasks [36–38]. Together, these studies suggest that profoundly important aspects of human cognition and behavior may be dismissed if the observed variability is averaged out and, therefore, not investigated.

Similarly, in attention and mind wandering research it has been shown that the behavioral success is linked to the successful dynamics of attentional resource allocation to task-related external demands as opposed to task-unrelated internal information [14,39]. In this study, we therefore combine this notion of resource allocation with response variability and apply it to the mind wandering problem and resulting observable behavioral correlates. We reason that for successful task performance prompt adaptation of the attentional system to the demands at hand is important and requires a mechanism that supports swift transitions.

Since dynamical systems operating near a critical state show the greatest propensity of producing swift transitions in the dynamics of the order parameter [40,41]—including temporal complexity [42]—, we hypothesize that a healthy attentional system may also operate close to a critical point [24,43,44]. This could allow it to display meaningful dynamics, related to the stability or variability of the resulting behavior. A key prediction derived from this hypothesis is that transitions in attention occur spontaneously [14], and that the resulting dynamics in the observable behavior will be characterized by trial-to-trial dependencies, long-range temporal correlations, as opposed to uncorrelated fluctuations, or no fluctuations at all. Further, the closer the transitions in attention are to the critical point (separating the ordered sub-critical and the disordered super-critical regime) the stronger the long-range temporal correlations in reaction-time fluctuations, in analogy to what has been reported for models of neuronal oscillations [32].

We investigate changes in behavioral dynamics through indirect performance probing in a sustained attention task and through direct experience sampling in a cued mind wandering task, and use the detrended fluctuation analysis (DFA) [45,46] to quantify the temporal complexity of reaction-time fluctuations. Further, motivated by the effects of mood on mind wandering [1,5], we show that mood has profound effects on these dynamics.

## Methods

### Participants

The participants were recruited at the VU Amsterdam, Dutch or English speaking with no history of neurological complications or substance abuse. Three experiments were performed (see details below), an attention- task with 62 participants (mean age = 25 (SD = 6.2), 32 Female) and an attention- and mood-induction task with 89 participants (mean age = 22.4 (SD = 2.5), 64 Female) and a cued mind wandering task with 35 participants (mean age = 21.3 (SD = 1.2), 25 Female). All participants signed the informed consent, the protocol was approved by the scientific and Ethical Review Board (VCWE) of the Faculty of Psychology and Education, VU Amsterdam.

### Overall study design

We conducted 3 independent experiments that are explained in detail below. In experiment 1, participants completed a 8 minutes sustained attention task (Continuous Temporal Expectancy Task (CTET), adapted from Connell et al., 2009)). In experiment 2, additional participants underwent a mood manipulation before participating in the same CTET paradigm. Only stimulus display times of the task were slightly longer. In experiment 3, additional participants completed a 12 minutes externally cued mind wandering task. All experiments were conducted in front of a computer screen in an isolated room.

#### Continuous Temporal Expectancy Task (CTET)

**Fig 1:**
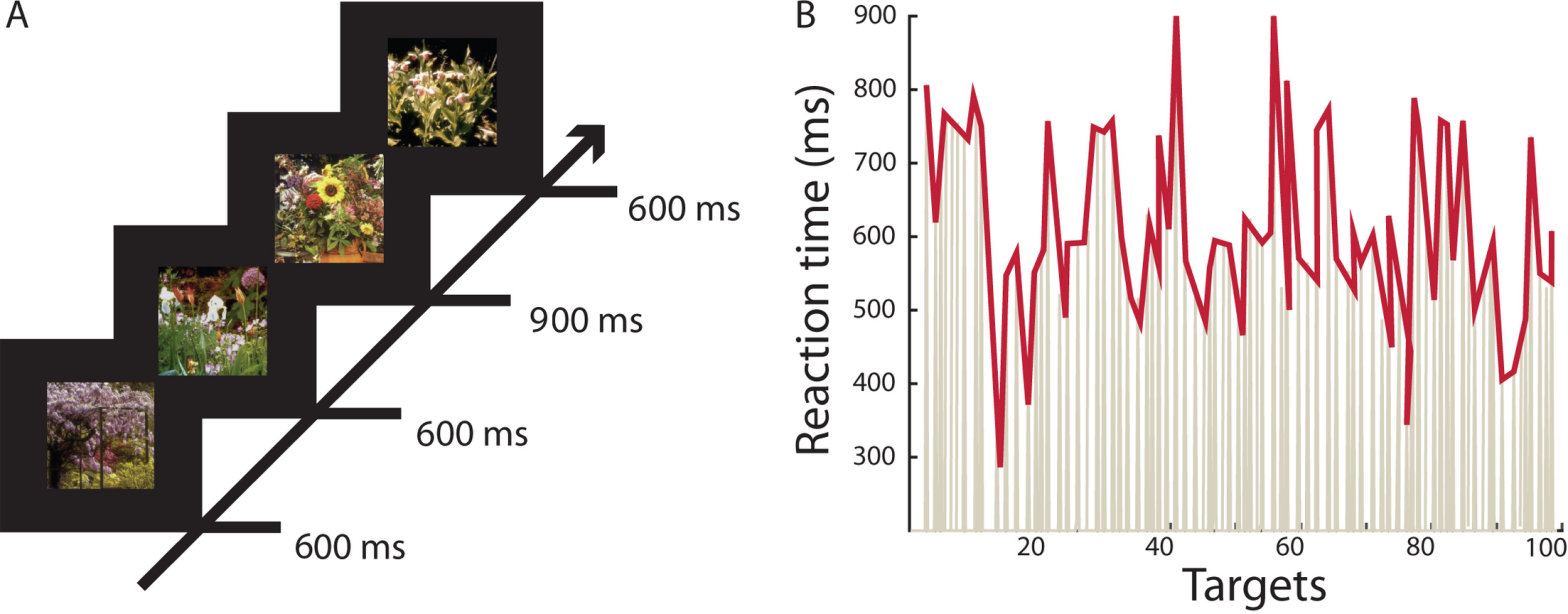
The Continuous Temporal Expectancy Task results in large variation in reaction times. (A) Illustration of the CTET paradigm (adapted from [44]) with stimuli presented for either 600 ms (900 ms experiment 2) if they were standard stimuli or for 1200 ms (1600 ms experiment 2) if they were targets. (B) Example sequence of reaction times exhibiting large variation to the 100 target images shown.

Participants completed a sustained attention task (adaptation of CTET [47]), which was designed to measure lapses in attention through reaction times and the errors that the participants make. The task consisted of centrally presented photos of flowers shown at regular intervals (600 ms in experiment 1, or 900 ms in experiment 2), resulting in a continuous stream of pictures. Participants were asked to attend to the temporal duration of each stimulus and press the space bar with their dominant hand when a stimulus was presented longer (1200 ms in experiment 1, or 1600 ms in experiment 2) than the standard duration (Fig 1). Long-duration stimuli occurred semi-randomly (every 4^th^ to 10^th^ stimuli) 100 times. Identifying the duration target was easy when fully attending to the stimuli; however, it quickly becomes demanding to fully focus on the boring task and results in great variation as well as occasional misses during the continuous task. This makes the CTET a measure of continuous deployment of attention to the time domain, i.e., time interval between events. The stimuli were made of naturalistic pictures taken from the International Affective Picture System [48], with pictures specifically chosen for their low arousal values. Additionally, the color, brightness, saturation, and size of the scenes were standardized decrease stimulus perceptional differences.

The participant’s ability to monitor the stimulus duration is increased when attention is actively oriented towards it [49], and decreases if top-down attentional effort is diminishing. Lapses in identifying targets may therefore been taken as a correlate of decreased attention, a phenomenon seen in many everyday life applications. Different from conceptually similar attention tasks such as the go/no-go “Sustained attention to response task” (SART [50]), every stimulus is a potential target and only discriminated by its longer presentation-time and not by perceptual features. This solves the problematic issue of target salience and automatically engaged exogenous attention interfering with the continuous attentional aspect which is targeted in this study [51]. In this study, a single block was chosen to tap into long-term sustained attention abilities of the participants.

#### Mood inductions

In experiment 2, before completing the CTET task the participants had two interventions to induce a positive, negative or neutral mood. They first watched one of three 5 minutes movies. The content of the ‘positive’ movie contained clips of funny sit-coms and playing puppies; the ‘negative’ movie contained clips from war scenes and the ‘neutral’ movie contained clips from a documentary on concrete pipes. The instruction was to pay special attention to the content of the movie, of which they would get a quiz at the end. This was meant to obscure the effect of the mood induction and to let the participants think this experiment was about memory, and not about the effect of mood on mind wandering and attention. The ethics committee approved withholding information about the purpose of the experiment from participants until the experiment was completed. Before and after the movie, the participants completed the Positive and Negative Affect Scale *(*PANAS, [52,53]) to verify successful mood manipulation. The PANAS comprises two mood scales, one for positive affect and one for negative. Participants indicate to what extend they feel certain emotions using a 5-point scale ranging from not at all (1) to extremely (5). The second mood induction was to bring a vivid memory in mind that makes them feel positive, negative or neutral, and then tell the test leader when they succeeded and vividly felt the emotions that come with this memory. Again, The PANAS was applied to check the success of the manipulation. Both times, the same mood was induced in the participant. At the end they were debriefed about the real purpose of the experiment.

*Externally cued mind wandering*: Probe-caught paradigms are popular means to investigate mind wandering (for review: [54]) and numerous studies have used thought probes during different tasks to index the degree of mind wandering at specific moments [13,18,55]. In our study, the participants sat in a dimly lit room with their eyes closed and were asked to focus their attention on the sensation of the breathing (bodily movements of the in- and exhalation) for 12 minutes. In semi-random intervals (jitter: 7–20 s, 100 probes in total) a tone asked them to indicate if they achieved that goal or if their mind had wandered away from the breath. Participants responded by promptly indicating the level of attention on a 3-point Likert scale (1- focused on the breath, 2- a little distracted, 3- totally absent).

#### Behavioral analysis

The observed reaction-time averages were calculated from the point in time when the target stimulus was displayed longer than non-target stimuli. The reaction time, therefore, includes both the time needed to notice the deviant and the time to react. The next stimulus is displayed after 600 ms (experiment 1) or 900ms (experiment 2), and to prevent that wrong presses to the non-targets stimuli would count as a very slow reaction to the target stimulus, we defined the maximum allowed reaction time up until 100 ms after the next stimuli was presented (700 ms experiment 1, 1000 ms experiment 2). To obtain a comprehensive reaction-time performance we included misses and in the reaction time series and assigned them the longest reaction time allowed (i.e., 700 ms or 1000 ms). Incorporating misses in the time-series avoids short average reaction times in subjects responding very fast but also missing several trials, and importantly, the temporal structure of an actual miss in the time series is preserved for the analysis of temporal correlations. Only serious task performances were taken into the analysis; therefore, we excluded participants who did not press the response key (Experiment 1: *n* = 2; Experiment 2: *n* = 1), or who pressed the same button in the entire experiment (Experiment 3: *n* = 1) from the analysis. For parametric tests paired-samples t-tests were used, with a significance level of *p* < 0.05. Associations between behavioral measures and long-range temporal correlations (LRTC) (see next section) were calculated using Pearson’s correlation coefficient.

#### Long-range temporal correlations analysis using the detrended fluctuation analysis

We were interested in understanding individual differences in reaction-time fluctuations as an indirect measure of fluctuations in mind wandering episodes. To this end, we quantified the strength of LRTC in reaction-time series using the detrended fluctuation analysis (DFA) [46]. The reaction-time series were defined by using the sequence of reaction times RT(*k*) with *k* being the index labeling the *k*’th reaction time from a total of *N* reaction times (*k* = 1, 2, …*N*). The k-index plays the role of a pseudo-time in the DFA. In brief, the DFA measures the power-law scaling of the root-mean-square fluctuation of the integrated and linearly detrended signals, *F*(*t*), as a function of time window size, *t* (with an overlap of 50% between windows). The DFA exponent (α) is the slope of the fluctuation function *F*(*t*) and can be related to the power-law scaling exponent of the auto-correlation function decay (γ) and the scaling exponent of the power spectrum density (β) by $\alpha =\frac{1+\;\beta }{2}=\frac{2-\gamma }{2}$ DFA exponent values between 0.5 and 1.0 reveal the presence of LRTC, whereas an uncorrelated signal has an exponent value of 0.5. The decay of temporal correlations was quantified over a range of 2 to 60 reaction times.

## Results

### Simulation for the behavioral analysis and theoretical framework

**Fig 2:**
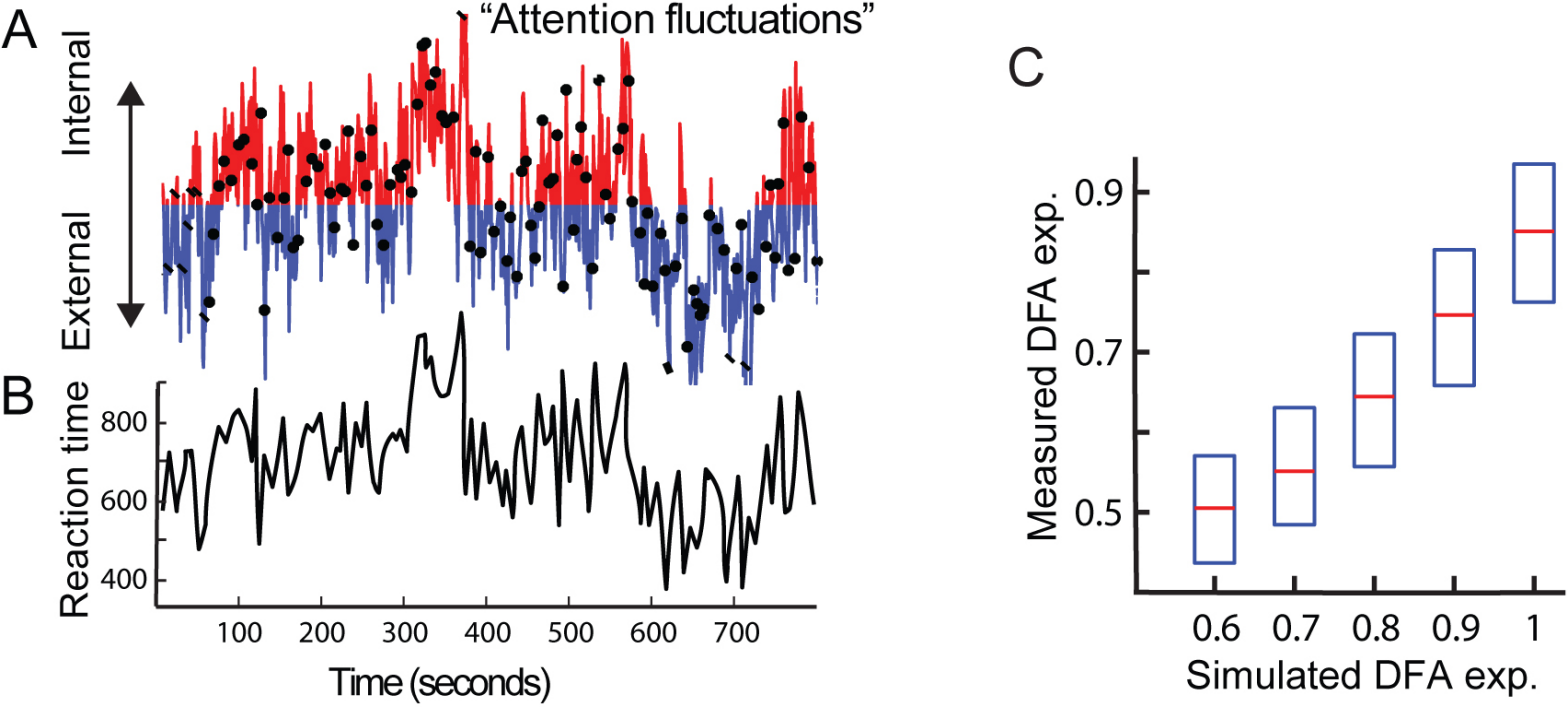
A model of attention fluctuations to explain non-random fluctuations in reaction times. (A) Our model is based on the hypothesis that attention fluctuates on a spectrum from highly external to highly internal with a non-random temporal structure, shown here for a DFA exponent of 0.8. The *black dots* indicate moments that target stimuli appear in the CTET experiment, which results in (B) a reaction-time series with a similar temporal structure under the assumption that reaction times are shorter when attention is strongly focused on external as opposed to internal sources of information. (C) 1/f signal produced with simulated sampling, showed a robust estimation of underlying temporal correlation with infrequent, semi-random sampling (*p* <.00001).

For the behavioral analysis, we work with the assumption that the attention system shows temporal fluctuations in form of LRTC. If these are reflected in behavioral performance over time, it should be possible to capture these dynamics with behavior samples during a sustained attention paradigm. Therefore, in our model, we first tested whether it was possible to quantify the hypothesized attentional dynamics with a low-frequency sampling rate, i.e., the occasional presentation of a target image in the CTET task. Therefore, before conducting the experiments, simulations were carried out to: 1. find the minimum amount of data points needed to reliably estimate DFA exponents (see Methods) and 2. see if the temporal correlations of an underlying signal can be recovered with the infrequent sampling used in the attention paradigm. For this, first a 1/f signal was produced in Matlab using a signal generator with defined underlying LRTC (DFA exponent in the range of 0.6 to 1). This temporal structure is representing the hypothesized fluctuations in attention over time on a scale from entirely focused on external environment to entirely focused on internally generated thoughts and feelings, commonly referred to as mind-wandering On top of that signal, time points were marked in accordance with the sampling frequency of the target presentation used in the sustained attention task (Fig 2A). The points of overlap (1/f signal* target presentation) were obtained, and the temporal structure of this “behavioral time series” was calculated (Fig 2B). For reliability of the recovery success, the underlying 1/f signal was simulated 100 times for each exponent of the underlying signal ranging from 0.6 to 1, and the DFA fluctuation function fitted from 2 to 60 events. The resulting comparison of original exponent with recovered exponent revealed that it is possible to recover the LRTC of an underlying time series with only 100 sampling points (*p*<.00001) (Fig 2C). Less than 100 samples were unfavorable as it led to a regression towards the mean with an underestimation of high and overestimation of low DFA, therefore decreasing the sensitivity of exponent recovery. Thus, the observed reaction-time series resulting from the task can be used as an estimation of the underlying temporal correlation of the participants’ attentional capacities.

### Result experiment 1: The temporal dynamics of reaction times are inversely related to performance

**Fig 3:**
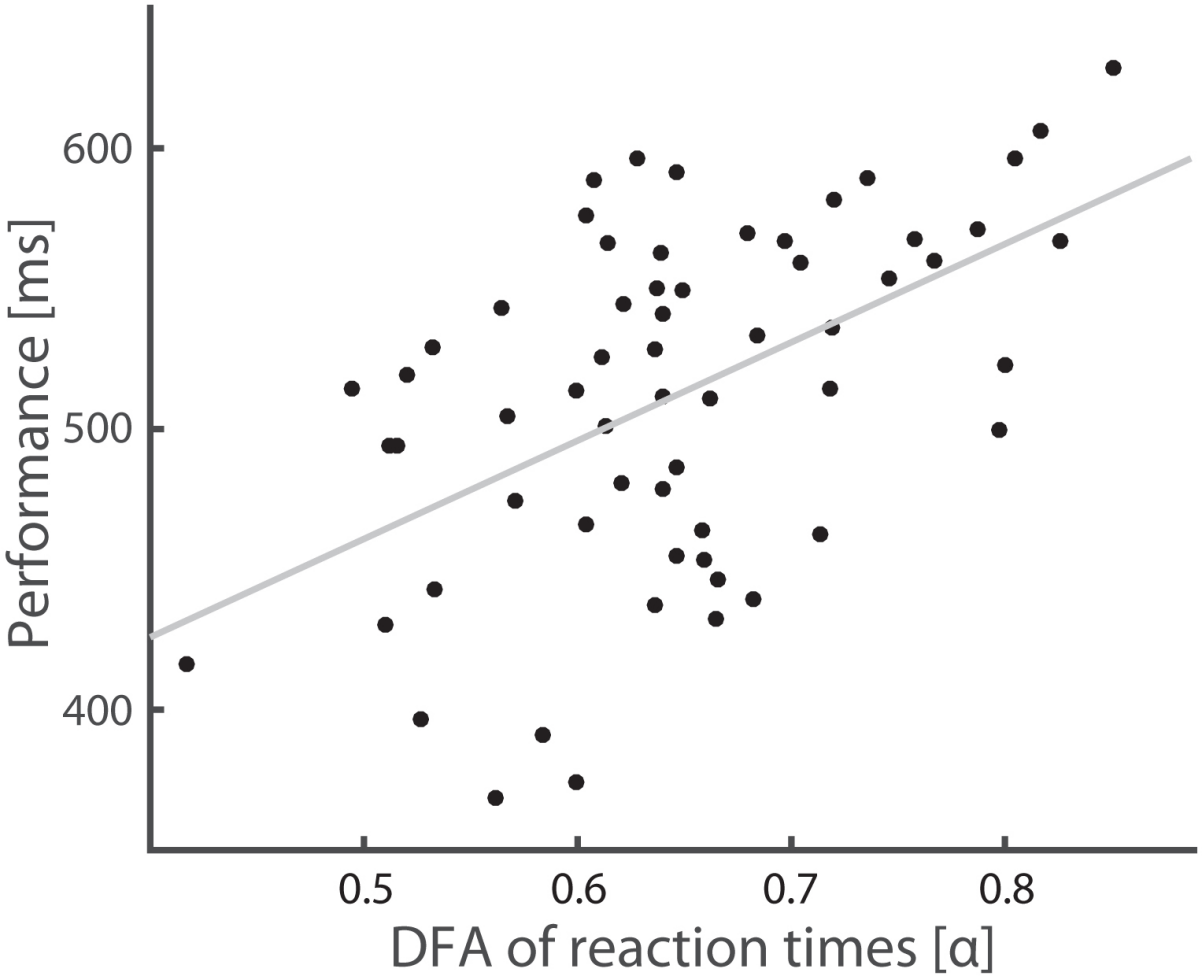
Weak LRTC of reaction-time series are associated with fast reaction times. The observed correlation (R^2^ = .52, *p* = .00002), shows that better performance is associated with less variability.

To test if the observed behavior during the sustained attention task exhibited LRTC, we computed the DFA exponent on the reaction time series (see Methods). Participants showed large individual variation in scaling exponents with a mean of α = .65 (SD = .09). Interestingly, DFA exponents correlated strongly and positively with the mean reaction time across subjects (R^2^ = .52, *p* = .00002), indicating that better performance was associated with a suppression of complex reaction-time fluctuations (Fig 3).

#### Control analysis

To verify that it was truly the temporal structure of the reaction time series that correlated with the mean reaction times, we randomly shuffled the reaction times and repeated the DFA analysis of the shuffled data. The correlation of DFA with mean reaction time disappeared (R^2^ = .03, *p* = .79). Additionally, after shuffling the mean DFA of all participants dropped from α = .65 to .55, as expected from a time series with a random temporal structure (see Methods).

### Results experiment 2: Mood manipulation

In experiment 2 we tested if it is possible to manipulate the dynamics observed in Experiment 1 with a positive, neutral or negative mood induction. To test the success one-way ANOVA’s were performed on the PANAS scores obtained before and after the first mood induction (movie) and after the second mood induction (imagination). The positive affect test of the PANAS showed that after the interventions the positive group was higher in positive affect (significant between-groups main effect after the movie (*F*(2) = 4,958, *p* < .01 and after imagination (*F*(2) = 15,346, *p* < .001, see Table 1). The negative affect test of the PANAS showed that after the interventions the negative group was higher in negative affect (significant between-groups main effect after the movie (*F*(2) = 7,186, *p* < .01), and imagination (*F*(2) = 26.888, *p* < .001, see Table 2). Taken the results together, we conclude that the mood induction was successful.

**Table 1:** Results PANAS (positive affect).

Positive Mood Manipulation Check	Neutral mood induction (N = 46)	Positive mood induction (N = 20)	Negative mood induction (N = 22)	One-way ANOVA *p* value	Tukey post hoc tests (neu-pos, neu-neg, pos-neg)
**Measurement1 (pre-movie)**	3.01	3.38	3.14		
**Measurement2 (post-movie)**	2.80	3.31	2.76	.009	.013, .970, .022
**Measurement3 (post-imagine)**	2.42	3.51	2.36	.000	.000, .964, .000

**Table 2:** Results PANAS (negative affect).

Negative Mood Manipulation Check	Neutral mood induction (N = 46)	Positive mood induction (N = 20)	Negative mood induction (N = 22)	One-way ANOVA *p* value	Tukey post hoc tests (neu-pos, neu-neg, pos-neg)
**Measurement1 (pre-movie)**	1.99	1.89	1.83		
**Measurement2 (post-movie)**	1.54	1.52	2.16	.001	.996, .002, .007
**Measurement3 (post-imagine)**	1.36	1.47	2.57	.000	.781, .000, .000

### Results experiment 2: Positive mood is associated with faster performance and reduced temporal complexity of reaction times

**Fig 4:**
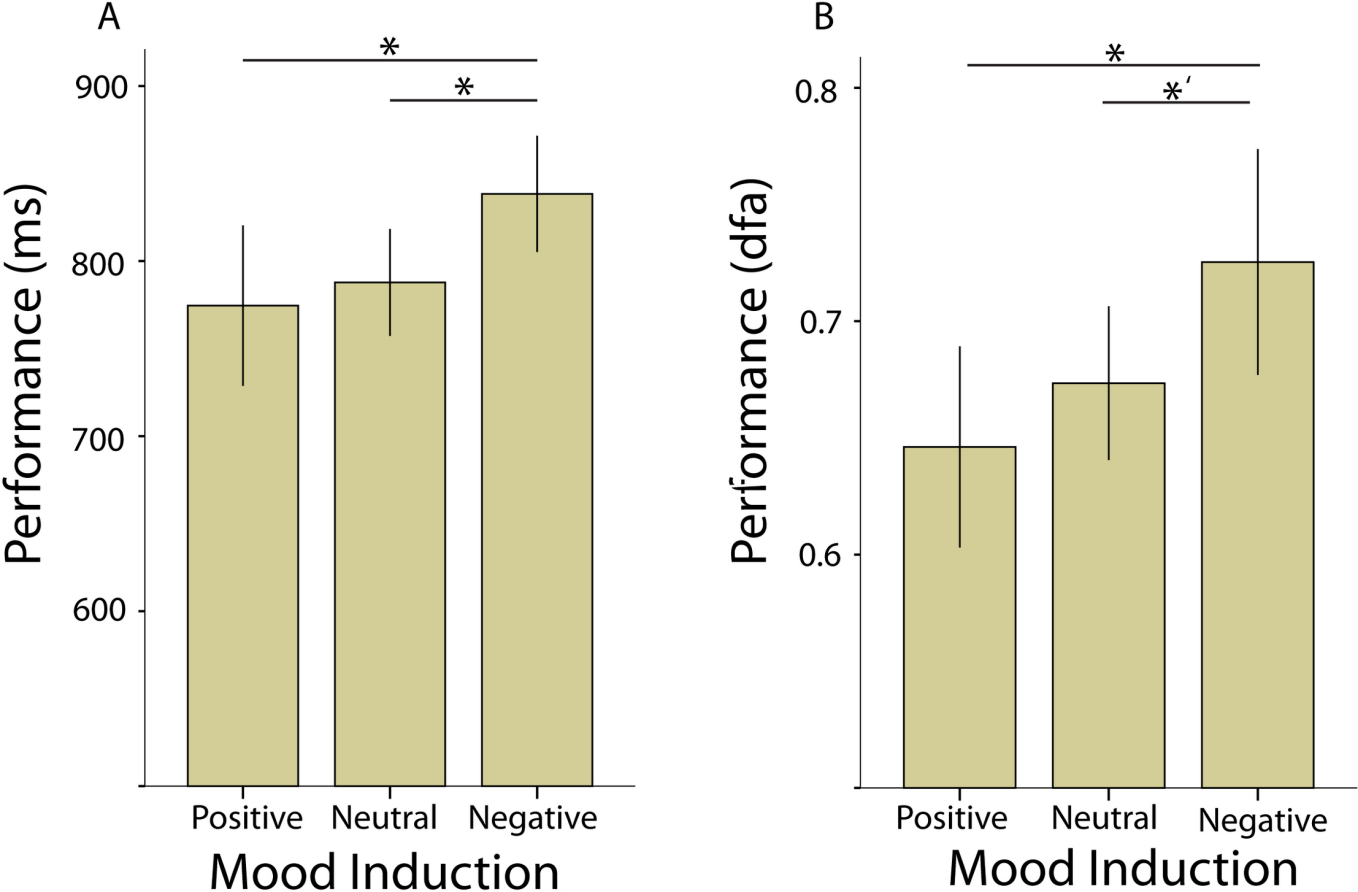
Mood has an effect on average reaction time and reaction-time temporal structure. Participants in the negative mood condition showed worse performnce than particiapnts in the neutral (t65 = 2.07, *p* = .042) and positve mood condition (t40 = 2.39, *p* = .022). Additionally, the temporal strucutre of reaction time series differed between positive—negative (t40 = 2.53, *p* = .016), Error bars represent 95% confidence intervals.

In experiment 2, we replicated the findings of experiment 1, and show that even with longer stimulus display times, the DFA exponents of the reaction times correlate strongly and positively with the mean reaction time across subjects (R^2^ = .56, *p* < 000001). Interestingly, dividing the outcome of the attention task in the three mood inductions showed that performance improves as the mood improves from a mean reaction time of 838 ms (SD = 75) in the negative condition to 788 ms (SD = 102) in the neutral (t65 = 2.07, *p* = .042) and to 774 ms (SD = 98) in the positive condition (t40 = 2.39, *p* = .022) (Fig 4A). Interestingly, not only the mean reaction times are influenced by mood but also the temporal structure of the behavioral time series. Negative mood was associated more complex variation in reation times (α = .73, SD = .11) compared to positve mood (α = .65, SD = .1; t40 = 2.53, *p* = .016), and a trend compared to neutral (α = .67, SD = .11; t65 = 1.9, *p* = .073), showing that mood also changed the variablity in the reaction time series (Fig 4B).

### Results experiment 3: Probe-caught mind wandering dynamics show LRTC

**Fig 5:**
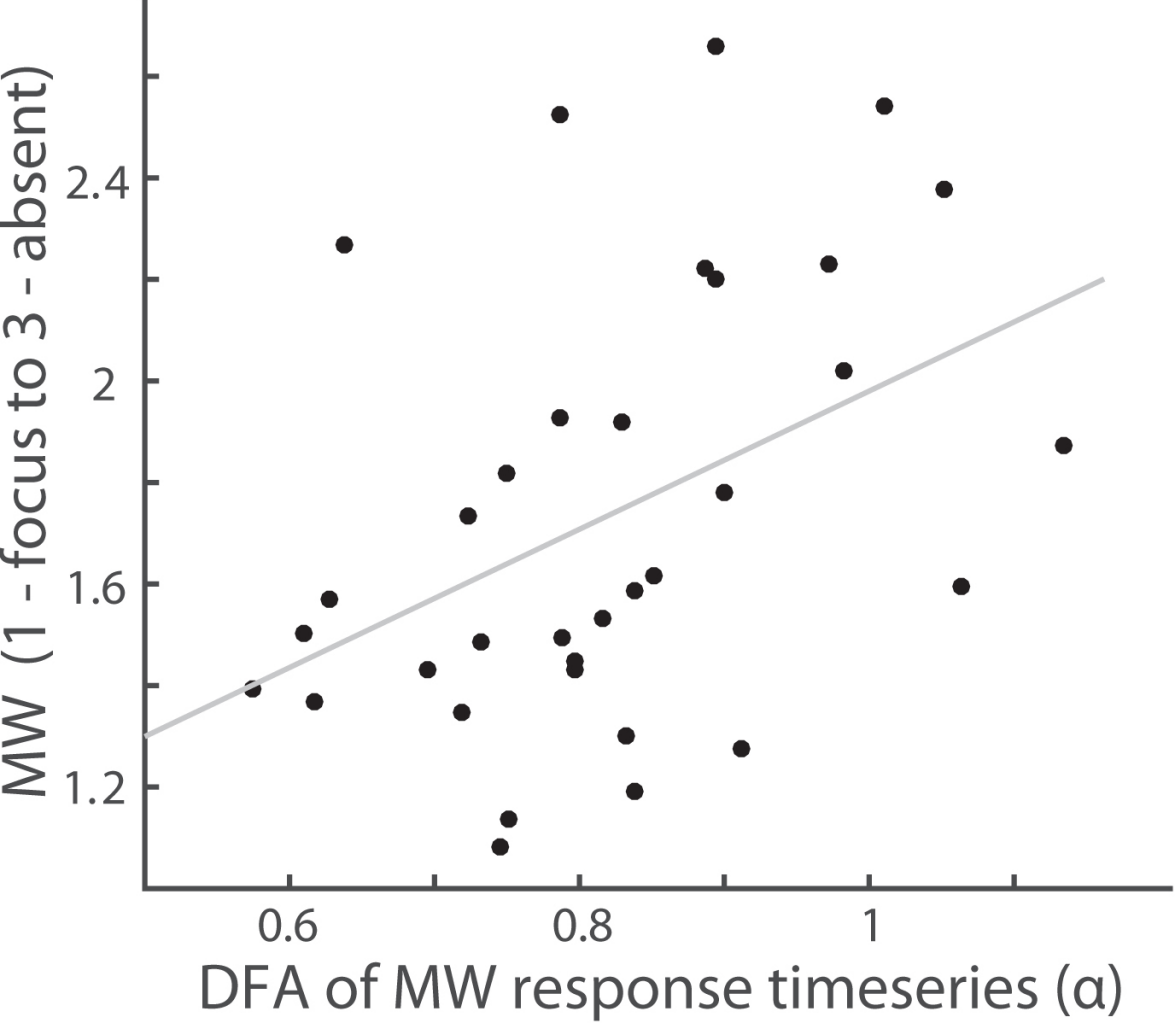
More mind wandering episodes are associated with increased LRTC of response-time series. The Correlation between DFA the of the subjective rating of attention, or mind wandering episodes shows that variability increases with more mind wandering (R^2^ = .43, *p* = .01).

Indirect probing in the form of sustained attention tasks has the limitation that it is unknown if the participants really had increased mind wandering or not. Therefore, we investigated if subjectively perceived mind wandering also shows these dynamics. Participants were asked to focus their attention on the sensation of the breathing and indicate the level of success upon probing on a 3-point Likert scale (1- focused on the breath, 2- a bit focused/a bit distracted, 3- totally absent/mind wandering). Missed responses were counted as totally absent (3). We found that the average score of attention over the entire experiment was 1.73 (SD = 0.43), showing that participants were able to focus on the breath, with occasional distractions. Importantly, we found that these mind wandering periods also exhibited long-range temporal correlations (average α = .82 (SD = .12)). These LRTC of responses are inversely related to mind wandering: the more often participants reply that they were mind wandering the higher the DFA, showing that also subjectively felt focus is associated with reduced temporal complexity (R^2^ = .43, *p* = .01) (Fig 5).

#### Control analysis

First, we tested the confidence of the participants in the accuracy of their mind wandering rating on a 5-point Likert scale (1- strongly disagree to 5- strongly agree). The average score of 3.88 (SD = 0.8) shows that participants believed their scores to be reflecting their subjective experience. Then, again we performed the control correlation analysis with randomly shuffled values (disrupting the original underlying temporal structure while keeping mean values the same), which showed that the correlation of DFA with mean response disappeared again (R^2^ = -.18, *p* = .30). Additionally, after shuffling the mean DFA of all participants reduced from α = .82 to .59, again approaching random temporal structure in the response series.

## Discussion

In this study, we tested the hypothesis that spontaneous transitions in attention can be characterized by LRTC. Assuming that a healthy attention system is adapted to allow self-organized switches in response to changing environmental (or internal) demands and, therefore, could operate near a critical state, we applied a measure of temporal correlations to behavior time series. Through indirect performance probing in a sustained attention task and direct experience sampling of mind wandering episodes, we showed that both behavioral time series display long-range temporal correlations. Further, we showed that better performance is associated with less complexity in the response variation. In addition, we could actively manipulate the temporal structure and performance by inducing different moods in the participants.

Investigations into attention and mind wandering often omit the ongoing variability displayed by the participants over time as noise or unexplained variance [16], and focusing instead on measures of central tendency such as mean reaction times [14,15]. In this study, we investigated if the variance caused by lapses in attention and mind-wandering episodes are meaningful and can be quantified with the use of scaling techniques to estimate LRTC, also known as *1/f* noise [16]. For this, we first show that it is possible to retrieve underlying LRTC in a signal with irregular sampling frequency (as used in our attention task) to allow behavioral correlates as a measure for attentional focus. To quantify the temporal complexity of the system we applied the detrended fluctuation analysis (DFA) [45,46], and achieved a robust estimation of underlying temporal correlation. We showed that the observed behavioral responses have non-random clustered fluctuations in the form of LRTC, as opposed to uncorrelated fluctuations, or no fluctuations at all.

According to our hypothesis, a healthy attention system—like many other physiological systems, including neuronal oscillations [28,29], operate near a critical state [24,43,44]. It is plausible that the fluctuations in focus of attention—and hence in the reaction times—may find an explanation also within the framework referred to as “rapid transition processes” [56], which has been successful in accounting for the temporal complexity of different sleep stages and levels of consciousness [57–59]. Importantly, the metric of LRTC applied to reaction-time series is efficient in identifying a direct relationship between individual differences in performance and temporal variability in performance. Therefore, we show that applied to sustained attention and mind wandering LRTC might be related to the dynamics of the allocation of limited attentional resources; possibly from task-unrelated (internal) demands such as mind wandering to task-related (or external) processes such as responding to the task. The closer the transitions in attention are to the critical point (separating the ordered sub-critical and the disordered super-critical regime), the stronger the long-range temporal correlations in the behavior. The closer a state is to criticality (higher LRTC) it may be more optimized for a broad *range* of different demands including transitions from environmental and internal sources [60], while reducing these fluctuations might be more beneficial for a single focus, as needed during the present attention task. Indeed, we also see a reduction in LRTC in neuronal oscillations in response to task performance [61,62]. A reduction in exponents indicates less autocorrelations and, therefore, less influence on future dynamics [63,64], hence possibly less distractions from the focused task at hand, such as off-task mind wandering.

It is important to stress that the control analysis showed that the temporal structure measured was not only caused by increased variance produced by the increase in reaction times, or mind-wandering episodes, but that additionally these episodes come with an increased temporal structure related to the success in performance. The latter is lost if the order of responses is shuffled: The reaction-time average and variability stay the same while the temporal structure decreases.

We know that mind wandering [1,5] and our ability to keep attention on a task can be largely influenced by our mood [8]. Therefore, we tested if we could actively manipulate the temporal structure of performance by inducing different moods in the participants. We show that indeed positive mood was associated with better performance and decreased LRTC. Bad mood is associated with increased mind wandering [1], with one reason being that we turn ‘inside’ in an attempt to gain insight into why we feel bad [65] a process accentuated by the observation that bad mood additionally biases the mind for further negative thinking [66]. In accordance with our hypothesis participants most likely were more often distracted or pulled out of the single external focus required for the sustained attention task to more internal mood-related mind wandering or distractions. Interestingly, these switches did not occur ‘at random’ but are temporally related.

### Outlook

The implications of these results stress the importance of future research to investigate the temporal structure of behavioral variability instead of only central tendency measurements. It is also interesting to apply neuroimaging techniques to understand the neural correlations of these individual differences in performance variability, as scaling techniques have successfully been applied to both EEG [62,67,68] and fMRI investigations [69,70].

## References

[1] Killingsworth MA, Gilbert DT. A wandering mind is an unhappy mind. Science. 2010 p. 932 doi: 10.1126/science.1192439 21071660 10.1126/science.1192439

[2] Buckner RL, Andrews-Hanna JR, Schacter DL. The brain’s default network: anatomy, function, and relevance to disease. Ann N Y Acad Sci. 2008;1124: 1–38. doi: 10.1196/annals.1440.011 18400922 10.1196/annals.1440.011

[3] Smallwood J, Brown K, Baird B, Schooler JW. Cooperation between the default mode network and the frontal-parietal network in the production of an internal train of thought. Brain Res. Elsevier B.V.; 2012;1428: 60–70. doi: 10.1016/j.brainres.2011.03.072 21466793 10.1016/j.brainres.2011.03.072

[4] He J, Becic E, Lee YC, McCarley JS. Mind wandering behind the wheel: Performance and oculomotor correlates. Hum Factors. 2011;53: 13–21. doi: 10.1177/0018720810391530 21469530 10.1177/0018720810391530

[5] Hamilton JP, Furman DJ, Chang C, Thomason ME, Dennis E, Gotlib IH. Default-mode and task-positive network activity in major depressive disorder: implications for adaptive and maladaptive rumination. Biol Psychiatry. Elsevier Inc.; 2011;70: 327–33. doi: 10.1016/j.biopsych.2011.02.003 21459364 10.1016/j.biopsych.2011.02.003 PMC3144981

[6] Papageorgiou C, Wells A. An empirical test of a clinical metacognitive model of rumination and depression Cognit Ther Res. Springer; 2003;27: 261–273.

[7] Mellings TMB, Alden LE. Cognitive processes in social anxiety: the effects of self-focus, rumination and anticipatory processing. Behav Res Ther. 2000;38: 243–257. doi: 10.1016/S0005-7967(99)00040-6 10665158 10.1016/s0005-7967(99)00040-6

[8] Lyubomirsky S, Kasri F, Zehm K. Dysphoric rumination impairs concentration on academic tasks. Cognit Ther Res. 2003;27: 309–330. doi: 10.1023/A:1023918517378

[9] Driver J. A selective review of selective attention research from the past century. Br J Psychol. 2001;92 Part 1: 53–78. doi: 10.1348/000712601162103 11256770

[10] Sarter M, Gehring WJ, Kozak R. More attention must be paid: The neurobiology of attentional effort. Brain Research Reviews. 2006 pp. 145–160. doi: 10.1016/j.brainresrev.2005.11.002 16530842 10.1016/j.brainresrev.2005.11.002

[11] Theeuwes J. Top-down and bottom-up control of visual selection. Acta Psychol (Amst). Elsevier B.V.; 2010;135: 77–99. doi: 10.1016/j.actpsy.2010.02.006 20507828 10.1016/j.actpsy.2010.02.006

[12] Smallwood J, Schooler JW. The Science of Mind Wandering: Empirically Navigating the Stream of Consciousness. Annu Rev Psychol. 2015;66: 487–518. doi: 10.1146/annurev-psych-010814-015331 25293689 10.1146/annurev-psych-010814-015331

[13] Seli P, Cheyne JA, Smilek D. Wandering minds and wavering rhythms: Linking mind wandering and behavioral variability. J Exp Psychol Hum Percept Perform. 2013;39: 1–5. doi: 10.1037/a0030954 23244046 10.1037/a0030954

[14] Kelly a MC, Uddin LQ, Biswal BB, Castellanos FX, Milham MP. Competition between functional brain networks mediates behavioral variability. Neuroimage. 2008;39: 527–37. doi: 10.1016/j.neuroimage.2007.08.008 17919929 10.1016/j.neuroimage.2007.08.008

[15] Diniz A, Wijnants ML, Torre K, Barreiros J, Crato N, Bosman AMT, et al Contemporary theories of 1/f noise in motor control. Hum Mov Sci. Elsevier B.V.; 2011;30: 889–905. doi: 10.1016/j.humov.2010.07.006 21196059 10.1016/j.humov.2010.07.006

[16] Gilden DL. Cognitive emissions of 1/f noise. Psychol Rev. 2001;108: 33–56. doi: 10.1037/0033-295X.108.1.33 11212631 10.1037/0033-295x.108.1.33

[17] Kane MJ, McVay JC. What Mind Wandering Reveals About Executive-Control Abilities and Failures. Curr Dir Psychol Sci. 2012;21: 348–354. doi: 10.1177/0963721412454875

[18] Smallwood J, Beach E, Schooler JW, Handy TC. Going AWOL in the brain: mind wandering reduces cortical analysis of external events. J Cogn Neurosci. 2008;20: 458–69. doi: 10.1162/jocn.2008.20037 18004943 10.1162/jocn.2008.20037

[19] Bastian M, Sackur J. Mind wandering at the fingertips: Automatic parsing of subjective states based on response time variability. Front Psychol. 2013;4 doi: 10.3389/fpsyg.2013.00573 24046753 10.3389/fpsyg.2013.00573 PMC3763218

[20] Esterman M, Noonan SK, Rosenberg M, Degutis J. In the zone or zoning out? Tracking behavioral and neural fluctuations during sustained attention. Cereb Cortex. 2013;23: 2712–2723. doi: 10.1093/cercor/bhs261 22941724 10.1093/cercor/bhs261

[21] VERPLANCK WS, Collier GH, COTTON JW. Nonindependence of successive responses in measurements of the visual threshold. J Exp Psychol. Not Available; 1952;44: 273–82. doi: 10.1037/h0054948 13000069 10.1037/h0054948

[22] Wertheimer M. An investigation of the “randomness” of threshold measurements. J Exp Psychol. 1953;45: 294–303. doi: 10.1037/h0055277 13052865 10.1037/h0055277

[23] Hardstone R, Poil SS, Schiavone G, Jansen R, Nikulin V V, Mansvelder HD, et al Detrended fluctuation analysis: A scale-free view on neuronal oscillations. Front Physiol. Frontiers Media SA; 2012;3 NOV. doi: 10.3389/fphys.2012.00450 23226132 10.3389/fphys.2012.00450 PMC3510427

[24] Bak P. Complexity and Criticality How nature works. Springer; 1996 pp. 1–32.

[25] Goldberger A. L., Amaral L. a N., Hausdorff J. M, Ivanov P. C, Peng C.-K, & Stanley HE, Goldberger AL, Amaral L a N, Hausdorff JM, Ivanov PC, Peng C-K, et al Fractal dynamics in physiology: alterations with disease and aging. Proc Natl Acad Sci U S A. 2002;99 Suppl 1: 2466–72. doi: 10.1073/pnas.012579499 11875196 10.1073/pnas.012579499 PMC128562

[26] Daliri MR. Automatic diagnosis of neuro-degenerative diseases using gait dynamics. Measurement. 2012;45: 1729–1734. doi: 10.1016/j.measurement.2012.04.013

[27] Voss RF, Clarke J. 1/F Noise in Music and Speech. Nature. 1975 pp. 317–318. doi: 10.1038/258317a0

[28] Montez T, Poil S-S, Jones BF, Manshanden I, Verbunt JPA, van Dijk BW, et al Altered temporal correlations in parietal alpha and prefrontal theta oscillations in early-stage Alzheimer disease. PNAS. 2009;106: 1614–1619. doi: 10.1073/pnas.0811699106 19164579 10.1073/pnas.0811699106 PMC2635782

[29] Palva S, Linkenkaer-Hansen K, Näätänen R, Palva JM. Early neural correlates of conscious somatosensory perception. J Neurosci. 2005;25: 5248–58. doi: 10.1523/JNEUROSCI.0141-05.2005 15917465 10.1523/JNEUROSCI.0141-05.2005 PMC6724814

[30] Eguiluz VM, Chialvo DR, Cecchi G a., Baliki M, Apkarian a. V. Scale-free brain functional networks. 2003; 1–4. doi: 10.1103/PhysRevLett.94.018102 15698136 10.1103/PhysRevLett.94.018102

[31] van Orden GC, Kello CT, Holden JG. Situated behavior and the place of measurement in psychological theory. Ecol Psychol. 2010;22: 24–43. doi: 10.1080/10407410903493145

[32] Poil S-S, Hardstone R, Mansvelder HD, Linkenkaer-Hansen K. Critical-state dynamics of avalanches and oscillations jointly emerge from balanced excitation/inhibition in neuronal networks. J Neurosci. 2012;32: 9817–9823. doi: 10.1523/JNEUROSCI.5990-11.2012 22815496 10.1523/JNEUROSCI.5990-11.2012 PMC3553543

[33] Monto S, Palva S, Voipio J, Palva JM. Very slow EEG fluctuations predict the dynamics of stimulus detection and oscillation amplitudes in humans. J Neurosci. 2008;28: 8268–8272. doi: 10.1523/JNEUROSCI.1910-08.2008 18701689 10.1523/JNEUROSCI.1910-08.2008 PMC6670577

[34] Palva JM, Zhigalov A, Hirvonen J, Korhonen O, Linkenkaer-Hansen K, Palva S. Neuronal long-range temporal correlations and avalanche dynamics are correlated with behavioral scaling laws. Proc Natl Acad Sci U S A. National Acad Sciences; 2013;110: 3585–90. doi: 10.1073/pnas.1216855110 23401536 10.1073/pnas.1216855110 PMC3587255

[35] Kello CT, Anderson GG, Holden JG, Van Orden GC. The pervasiveness of 1/f scaling in speech reflects the metastable basis of cognition. Cogn Sci. 2008;32: 1217–1231. doi: 10.1080/03640210801944898 21585450 10.1080/03640210801944898

[36] Torre K, Balasubramaniam R, Rheaume N, Lemoine L, Zelaznik HN. Long-range correlation properties in motor timing are individual and task specific. 2011; doi: 10.3758/s13423-011-0049-1 21327380 10.3758/s13423-011-0049-1

[37] Smit DJA, Linkenkaer-Hansen K, Geus EJC de. Long-Range Temporal Correlations in Resting-State Alpha Oscillations Predict Human Timing-Error Dynamics. J Neurosci. 2013;33: 11212–11220. doi: 10.1523/JNEUROSCI.2816-12.2013 23825424 10.1523/JNEUROSCI.2816-12.2013 PMC6618606

[38] Kello CT, Beltz BC, Holden JG, Van Orden GC. The emergent coordination of cognitive function. J Exp Psychol Gen. 2007;136: 551–68. doi: 10.1037/0096-3445.136.4.551 17999570 10.1037/0096-3445.136.4.551

[39] Weissman DH, Roberts KC, Visscher KM, Woldorff MG. The neural bases of momentary lapses in attention. Nat Neurosci. 2006;9: 971–8. doi: 10.1038/nn1727 16767087 10.1038/nn1727

[40] Contoyiannis YF, Diakonos FK. Criticality and intermittency in the order parameter space. Phys Lett Sect A Gen At Solid State Phys. 2000;268: 286–292. doi: 10.1016/S0375-9601(00)00180-8

[41] Contoyiannis YF, Diakonos FK, Malakis A. Intermittent dynamics of critical fluctuations. Phys Rev Lett. APS; 2002;89: 35701. 10.1103/PhysRevLett.89.035701 12144402

[42] Turalska M, West BJ, Grigolini P. Temporal complexity of the order parameter at the phase transition. Phys Rev E. APS; 2011;83: 61142. 10.1103/PhysRevE.83.061142 21797337

[43] Kinouchi O, Copelli M. Optimal dynamical range of excitable networks at criticality. Nat Phys. 2006;2: 348–351. doi: 10.1038/nphys289

[44] Shew WL, Yang H, Petermann T, Roy R, Plenz D. Neuronal avalanches imply maximum dynamic range in cortical networks at criticality. J Neurosci. 2009;29: 15595–600. doi: 10.1523/JNEUROSCI.3864-09.2009 20007483 10.1523/JNEUROSCI.3864-09.2009 PMC3862241

[45] Linkenkaer-Hansen K, Nikouline V V, Palva JM, Ilmoniemi RJ. Long-range temporal correlations and scaling behavior in human brain oscillations. J Neurosci. 2001;21: 1370–7. 11160408 10.1523/JNEUROSCI.21-04-01370.2001 PMC6762238

[46] Peng K, Havlin S., Stanley H., and. Goldberger A. Quantification of scaling exponents and crossover phenomena in nonstationary heartbeat time series. Chaos. 1995;5. 10.1063/1.166141 11538314

[47] O’Connell RG, Dockree PM, Robertson IH, Bellgrove MA, Foxe JJ, Kelly SP. Uncovering the Neural Signature of Lapsing Attention: Electrophysiological Signals Predict Errors up to 20 s before They Occur. J Neurosci. 2009;29: 8604–8611. doi: 10.1523/JNEUROSCI.5967-08.2009 19571151 10.1523/JNEUROSCI.5967-08.2009 PMC6665670

[48] Lang P. J., Bradley M. M., & Cuthbert B. N. International affective picture system (IAPS): Technical manual and affective ratings. NIMH Center for the Study of Emotion and Attention. 1997 39–58.

[49] Nobre A, Correa A, Coull J. The hazards of time. Current Opinion in Neurobiology. 2007 pp. 465–470. doi: 10.1016/j.conb.2007.07.006 17709239 10.1016/j.conb.2007.07.006

[50] Robertson IH, Manly T, Andrade J, Baddeley BT, Yiend J. “Oops!”: Performance correlates of everyday attentional failures in traumatic brain injured and normal subjects. Neuropsychologia. 1997;35: 747–758. doi: 10.1016/S0028-3932(97)00015-8 9204482 10.1016/s0028-3932(97)00015-8

[51] Robertson IH, O’Connell R. Vigilant attention. Attention and Time. 2012 doi: 10.1093/acprof:oso/9780199563456.003.0006

[52] Engelen U, Peuter S De, Victoir A, Diest I Van, Van den Bergh O. Verdere validering van de Positive and Negative Affect Schedule (PANAS) en vergelijking van twee Nederlandstalige versies Psychol Gezondh. Springer; 2006;34: 61–70.

[53] Watson D, Clark LA, Tellegen A. Development and Validation of Brief Measures of Positive and Negative Affect. The PANAS Scales. 1988;54: 1063‐1070. doi: 10.1037/0022-3514.54.6.1063 10.1037//0022-3514.54.6.1063 3397865

[54] Smallwood J, Schooler JW. The Restless Mind. Psychol Bull. 2006;132: 946–958. doi: 10.1037/0033-2909.132.6.946 17073528 10.1037/0033-2909.132.6.946

[55] Christoff K, Gordon AM, Smallwood J, Smith R, Schooler JW. Experience sampling during fMRI reveals default network and executive system contributions to mind wandering. Proc Natl Acad Sci U S A. 2009;106: 8719–24. doi: 10.1073/pnas.0900234106 19433790 10.1073/pnas.0900234106 PMC2689035

[56] Kaplan AY, Fingelkurts AA, Fingelkurts AA, Borisov S V., Darkhovsky BS. Nonstationary nature of the brain activity as revealed by EEG/MEG: Methodological, practical and conceptual challenges. Signal Processing. 2005 pp. 2190–2212. doi: 10.1016/j.sigpro.2005.07.010

[57] Paradisi P, Allegrini P, Gemignani A, Laurino M, Menicucci D, Piarulli A. Scaling and intermittency of brain events as a manifestation of consciousness. AIP Conference Proceedings. 2013 doi: 10.1063/1.4776519

[58] Allegrini P, Paradisi P, Menicucci D, Laurino M, Bedini R, Piarulli A, et al Sleep unconsciousness and breakdown of serial critical intermittency: New vistas on the global workspace. Chaos, Solitons and Fractals. 2013; doi: 10.1016/j.chaos.2013.05.019

[59] Allegrini P, Paradisi P, Menicucci D, Laurino M, Piarulli A, Gemignani A. Self-organized dynamical complexity in human wakefulness and sleep: Different critical brain-activity feedback for conscious and unconscious states. Phys Rev E—Stat Nonlinear, Soft Matter Phys. 2015; doi: 10.1103/PhysRevE.92.032808 26465529 10.1103/PhysRevE.92.032808 PMC4909144

[60] Beggs JM, Plenz D. Neuronal Avalanches in Neocortical Circuits. J Neurosci. 2003;23: 11167–11177. doi:23/35/11167 [pii] 14657176 10.1523/JNEUROSCI.23-35-11167.2003 PMC6741045

[61] He BJ, Zempel JM, Snyder AZ, Raichle ME. The temporal structures and functional significance of scale-free brain activity. Neuron. Elsevier Ltd; 2010;66: 353–369. doi: 10.1016/j.neuron.2010.04.020 20471349 10.1016/j.neuron.2010.04.020 PMC2878725

[62] Irrmischer M, Sangiuliano Intra F, Mansvelder HD, Poil S-S, Linkenkaer-Hansen K. Strong long-range temporal correlations of beta/gamma oscillations are associated with poor sustained visual attention performance. Eur J Neurosci. 2017;38: 42–49. doi: 10.1111/ejn.13672 28858404 10.1111/ejn.13672 PMC6221163

[63] Eke a, Herman P, Kocsis L, Kozak LR. Fractal characterization of complexity in temporal physiological signals. Physiol Meas. 2002;23: R1–R38. doi: 10.1088/0967-3334/23/1/201 11876246 10.1088/0967-3334/23/1/201

[64] Mandelbrot BB, Van Ness JW. Fractional Brownian Motions, Fractional Noises and Applications. SIAM Rev. 1968;10: 422–437. doi: 10.1137/1010093

[65] Lyubomirsky S, Nolen-Hoeksema S. Self-perpetuating properties of dysthoric rumination. [Internet]. Journal of Personality and Social Psychology. 1993 pp. 339–349. doi: 10.1037/0022-3514.65.2.339 8366423 10.1037//0022-3514.65.2.339

[66] Koster EHW, De Raedt R, Goeleven E, Franck E, Crombez G. Mood-Congruent Attentional Bias in Dysphoria: Maintained Attention to and Impaired Disengagement From Negative Information. Emotion. 2005;5: 446–455. doi: 10.1037/1528-3542.5.4.446 16366748 10.1037/1528-3542.5.4.446

[67] Allegrini P, Menicucci D, Bedini R, Fronzoni L, Gemignani A, Grigolini P, et al Spontaneous brain activity as a source of ideal 1/f noise. Phys Rev E. APS; 2009;80: 61914. 10.1103/PhysRevE.80.061914 20365197

[68] Allegrini P, Paradisi P, Menicucci D, Gemignani A. Fractal complexity in spontaneous EEG metastable-state transitions: New vistas on integrated neural dynamics. Front Physiol. 2010; doi: 10.3389/fphys.2010.000128 10.3389/fphys.2010.00128 PMC3059954 21423370

[69] Tagliazucchi E, Balenzuela P, Fraiman D, Chialvo DR. Criticality in Large-Scale Brain fMRI Dynamics Unveiled by a Novel Point Process Analysis. Front Physiol. 2012;3: 1–12. doi: 10.3389/fphys.2012.00001 22347863 10.3389/fphys.2012.00015 PMC3274757

[70] Tagliazucchi E, Siniatchkin M, Laufs H, Chialvo DR. The voxel-wise functional connectome can be efficiently derived from co-activations in a sparse spatio-temporal point-process. Front Neurosci. 2016; doi: 10.3389/fnins.2016.00381 27601975 10.3389/fnins.2016.00381 PMC4994538

